# Early Detection of Autism Spectrum Disorder Through Automated Machine Learning

**DOI:** 10.3390/diagnostics15151859

**Published:** 2025-07-24

**Authors:** Khafsa Ehsan, Kashif Sultan, Abreen Fatima, Muhammad Sheraz, Teong Chee Chuah

**Affiliations:** 1 Department of Software Engineering, Bahria University Islamabad, Islamabad 44000, Pakistan; kehsan1997@gmail.com (K.E.); kashif.buic@bahria.edu.pk (K.S.); 2 Department of Professional Psychology, National University of Modern Languages, Islamabad 44000, Pakistan; ambermza@yahoo.com; 3 Centre for Wireless Technology, Faculty of Engineering, Multimedia University, Cyberjaya 63100, Selangor, Malaysia; engr.msheraz@gmail.com

**Keywords:** autism spectrum disorder, AUTOML, TPOT

## Abstract

**Background/Objectives**: Autism spectrum disorder (ASD) is a neurodevelopmental disorder distinguished by an extensive range of symptoms, including reduced social interaction, communication difficulties and tiresome behaviors. Early detection of ASD is important because it allows for timely intervention, which significantly improves developmental, behavioral, and communicative outcomes in children. However, traditional diagnostic procedures for identifying autism spectrum disorder (ASD) typically involve lengthy clinical examinations, which can be both time-consuming and costly. This research proposes leveraging automated machine learning (AUTOML) to streamline the diagnostic process and enhance its accuracy. **Methods**: In this study, by collecting data from various rehabilitation centers across Pakistan, we applied a specific AUTOML tool known as Tree-based Pipeline Optimization Tool (TPOT) for ASD detection. Notably, this study marks one of the initial explorations into utilizing AUTOML for ASD detection. The experimentations indicate that the TPOT provided the best pipeline for the dataset, which was verified using a manual machine learning method. **Results**: The study contributes to the field of ASD diagnosis by using AUTOML to determine the likelihood of ASD in children at prompt stages of evolution. The study also provides an evaluation of precision, recall, and F1-score metrics to confirm the correctness of the diagnosis. The propose TPOT-based AUTOML framework attained an overall accuracy 78%, with a precision of 83%, a recall of 90%, and an F1-score of 86% for the autistic class. **Conclusions**: In summary, this research offers an encouraging approach to improve the detection of autism spectrum disorders (ASD) in children, which could lead to better results for affected individuals and their families.

## 1. Introduction

Autism, often mentioned to as autism spectrum disorder (ASD), enclosed a broad spectrum of symptoms, abilities, and levels of impairment or disability among those affected [1]. It is important to note that autism is not a sickness or disease; rather, it is a lifelong neurological developmental disorder that significantly impacts communication, social skills, and behavior [2,3,4]. ASD serves as an umbrella term for a group of neurodevelopmental conditions associated with dificts in public interactivity and communication, as well as compact or repetitive behavior patterns [5]. Social difficulties are a key aspect of many neurodevelopmental disorders, profoundly influencing individuals with ASD, regardless of their cognitive or verbal capabilities [6]. According to the Centers for Disease Control and Prevention (2011), ASD is defined as a characterized by impaired development in social, linguistic and behavioral areas. This disablement often becomes apparent before the age of three and is typically associated with irregularity in intellectual abilities, learning, awareness, and sensory operations.

ASD often goes unnoticed in toddlers until a certain age, although symptoms usually appear within the first five years of life [7]. However, it can be diagnosed as early as 18 months old to two years [8,9,10]. The root causes of ASD have not yet been identified by scientists [11], but its causes can be genetic, with either parent or any other family member having the disorder, or resulting from any kind of complications during pregnancy [2,3,12]. Although some earlier hypotheses suggested a link between vaccine exposure and ASD, extensive evidence, including meta-analyses and epidemiological studies, has consistently demonstrated no causal relationship.

According to statistics from the World Health Organization (WHO), roughly 1% of the world’s population is affected by autism during the first two years of life [3]. However, detecting ASD is a difficult task., as there are many other mental disorders with overlapping symptoms [13]. As its symptoms appear at an early age of three years and continue for the rest of the individual’s life [14,15], early detection is crucial in reducing the effects of ASD through therapy [12,15,16]. The only proven therapy is behavioral therapy, and it is most effective when started at an early age [17,18,19]. According to the DSM-5 classification, autism spectrum disorder (ASD) is classified into three stringency levels, depending on the measure of public communication difficulty, confined and repetitive behaviors.

- Level 1 (Requiring Support): Affected individuals may have significant difficulty initiating social interactions and may have problems with organization and planning, but they are often able to function independently with some support.
- Level 2 (Requiring Substantial Support): There are obvious deficits in verbal and non-verbal communication. Social disablement are more severe, and inflexible behavior occurs frequently, even with support.
- Level 3 (Requiring Very Substantial Support): Affected individuals exhibit significant difficulties with verbal and nonverbal communication and extreme resistance to change. Their restricted and repetitive conduct significantly impairs their ability to function in all domains.

These levels help guide clinicians and caregivers in designing tailored interventions and understanding the intensity of support required at different developmental stages and settings. ASD also encompasses a spectrum of presentations, including Autistic Disorder, Asperger’s Syndrome, and Pathological Demand Avoidance (PDA) [2]. Children with ASD may encounter a range of developmental and behavioral challenges, including speech delays, difficulty in forming social relationships, attention deficits, and heightened sensory sensitivities, as reported in various studies [20,21]. These difficulties further emphasize the importance of early and accurate diagnosis for timely therapeutic intervention.

Children with ASD often present with a variety of co-occurring clinical conditions that can complicate diagnosis and intervention. These include neurodevelopmental delays, such as speech and intellectual disabilities, neurological issues, like epilepsy, and behavioral comorbidities, such as ADHD. Additionally, medical complications, including sleep disturbances and gastrointestinal problems, are frequently observed. Understanding this spectrum of associated conditions is essential for developing effective and context-sensitive diagnostic tools.

ASD symptoms vary from one child to another, but they generally fall into three categories [4,20,21] as follows:

- Social impairment—such as sharing emotions, holding a conversation.
- Communication difficulties—can be verbal (expressed through language) or non-verbal (such as facial expressions, eye contact, and gestures).
- Repetitive and stereotyped behavior—repeating words or actions.

The symptoms of ASD are thought to arise from atypical brain development and connectivity, particularly in areas connected with social cognition, communication, and executive functions. Neuroimaging studies have shown disrupted connectivity between the frontal cortex, amygdala, and temporal lobes, which are critical for processing emotional and social information. Additionally, abnormalities in synaptic pruning, neurotransmitter systems, and early disruptions in neural circuit formation contribute to impairments in sensory processing, learning, and adaptive behavior. These neurological differences help explain the characteristic social, communicative, and behavioral symptoms noticed in persons with ASD.

Receiving a formal diagnosis of ASD typically involves a lengthy process. In the United Kingdom, for example, families often face a waiting time of more than three years [22]. Identifying ASD at an early stage offers significant advantages for both the child and their caregivers, potentially improving developmental outcomes and lowering the financial burden linked to late diagnosis [4,23,24]. Several studies have emphasized the importance of recognizing ASD symptoms promptly [25], as early intervention plays a crucial role in fostering better communication, language development, and overall quality of life for affected children [20,26,27]. However, due to the lack of local assessment diagnostic tools and limited speech therapy facilities, especially in rural areas, autistic children often do not receive rehabilitation until the age of seven when they enter compulsory schooling. Therefore, early intervention is needed through a speedy diagnosis process. Conventionally, the Autism Diagnostic Observation Schedule (ADOS) is worn to determine the difference between the behavior of a child with ASD and without ASD. The Autism Diagnostic Interview-Revised (ADI-R) and the Diagnostic and Statistical Manual of Mental Disorders, Fifth Edition Text Revision (DSM-5-TR) are recognized as reliable diagnostic tools [2,20].

Symptoms of ASD typically become noticeable through the second year of life (12 to 24 months), although in cases of more notable developmental delay, they may be recognized later. In case of severe developmental delay, these symptoms are noted later than 24 months, as the symptoms that appear at this age can be distinguished from the typical developmental characteristics of a child [16]. Due to the variability of symptoms, an official diagnosis of ASD is frequently postponed until around the age of four [3]. Several tools are available for ASD screening, including the Autism Spectrum Quotient (AQ), the Social Communication Questionnaire (SCQ), and the Modified Checklist for Autism in Toddlers (M-CHAT), Childhood Autism Rating Scale-2 (CARS-2) and Toddler and Early Childhood Autism Screening Tool (STAT) [28]. The goal of screening and monitoring is to identify children at stake of ASD or who have already been exposed to it at an early stage. People such as parents, guardian, teachers and people without special education or training can perform these screenings. Information from these processes can be helpful for professionals to understand the behavior and condition of children.

The present study will provide an initial step towards the early diagnosis of ASD within a few minutes, saving time and expenses associated with multiple pre-diagnosis clinic visits for child observation and clinical interviews with parents or caregivers. In fact, this will be the primary diagnosis as first awareness of ASD among parents and caregivers in general and can become an authentic source of early detection guidance in developmental and behavioral therapy practices.

Identifying ASD at an early stage allows timely intervention, which significantly enhances the individual’s quality of life with autism [22,29]. Several studies have utilized data obtained from different screening tools to detect autism using machine learning (ML) approaches. Among these tools, the Q-CHAT-10 questionnaire has been frequently applied for screening ASD in children [30,31,32].

Automated machine learning (AUTOML) is a materializing field of AI that focuses on automating different phases of the machine learning process. Specifically, it automates model selection, hyperparameter tuning, feature engineering, and even the process of finding optimal architecture in deep learning, among others. In the field of ASD detection, AUTOML plays a significant role, as it enables non-professionals in the field of AI to construct high-performing models. Moreover, it eases the process of model development and optimizes model parameters. In this way, the model’s generalization capability can be increased, which is crucial for robust ASD detection across diverse patient populations. The workflow of AUTOML consists of on following stages: data preprocessing, model selection, hyperparameter tuning, ensemble tuning, and automated reporting [33,34].

To our knowledge, this study is one of the first to a TPOT-based AUTOML framework for ASD observation using real-world data collected from the rehabilitation centers across Pakistan.Unlike prior work that relies primarily on publicly available datasets from high-income countries, our study focuses on a locally developed dataset reflecting regional demographics and clinical practices. This approach offers novel insights into ASD detection in a culturally and contextually relevant population, helping bridge a reasonable breach in the literature.

The purpose of this study is to utilize AUTOML tools to identify whether a child is prone to ASD at the very early stages. The primary contributions of this study are as follows:

1. To gather empirical data for our study, we conducted a survey utilizing the autism spectrum quotient in toddlers-10 (Q-CHAT-10) questionnaire across various rehabilitation centers in Pakistan. The questionnaire comprises ten items that capture behavioral characteristics associated with ASD and seven other variables, including demographic information (e.g., age, gender). After administering the survey, the resulting dataset was obtained.
2. After collecting the dataset, we implemented an automated machine learning (AUTOML) pipeline using the TPOT (Tree-based Pipeline Optimization Tool) library for ASD detection. The TPOT automated the process of model selection and hyperparameter optimization.
3. In order to ensure the validity of the TPOT on our dataset, we conducted a thorough verification process. This involved manually recreating the machine learning model using the exact parameters generated by the TPOT. By replicating the model creation manually, we aimed to verify the consistency and reliability of TPOT’s automated pipeline. To examine and compute the effectiveness of both the AUTOML generated and manually recreated model, we employed a rigorous comparative evaluation.
4. Unlike manual model tuning, the TPOT automates the selection and optimization of ML pipelines through genetic programming, enabling the discovery of high-performing models with minimal human intervention. This makes it especially useful in healthcare scenarios where domain experts may not have ML expertise.

Our study introduces a novel framework that integrates domain-specific ASD screening data with automated machine learning (AUTOML) techniques to enable efficient and accessible early detection. The TPOT-based AUTOML tool automatically selects, optimizes, and evaluates pipelines using genetic programming, which is particularly beneficial in healthcare contexts where machine learning expertise is limited. By validating our model on a localized Pakistani dataset, the proposed system supports rapid and cost-effective ASD screening in low-resource clinical environments, making a unique contribution to both the fields of digital health and AI-assisted diagnostics in underserved regions.

The residue of the paper is structured as follow. The “Introduction” section includes the introduction of the study. The “Literature Review” section summarizes the previous works on ML that are related to ASD. The “Propose framework” section explains the functionality and methodology of the AUTOML system that we suggested and its implementation. The “Results and Evaluation” section shows the deduction and results obtained. Section 5 shows a comparison of the AUTOML -generated model with classical machine learning models; further, this section also compares the process of manual diagnosis with AUTOML -driven diagnosis. Section 6 is the discussion section. Finally, Section 7 highlights the conclusions and future plans to extend this work.

## 2. Literature Review

This section dispenses the synopsis of preceding research conducted in this field. Typically, for ASD diagnosis, standardized clinical tests are the only methods; they require hours of clinical assessments and a significant medical cost for diagnosis. Various techniques have been applied for ASD diagnosis, such as eye tracking techniques, brain imaging techniques, kinematic analysis, and so on. Numerous studies have used ML algorithms for either confirming a diagnosis or making an early ASD diagnosis.

Vakadkar et al. [1] proposed ML models for autism detection. The aim of this research was to discover whether a child is susceptible to autism during the initial stages of development. They designed an automated ASD prediction model to speed up the diagnosis compared to traditional methods. For this purpose, they applied the support vector machine, random forest classifier, naive Bayes, logistic regression, and k-nearest neighbor to a dataset compiled by Dr. Fadi Thabtah. This dataset was based on Q-CHAT-10 with 18 attributes and 1054 instances. Logistic regression gave the utmost accuracy. However, a constraint of this approach was that the dataset had a limited number of attributes and instances.

Erkan et al. [7] utilized three datasets (AQ-10-Adult, AQ-10-Adolescence, AQ-10-Child) from the UCI database for their research. The aim of the study was to offer a simplified approach to ASD diagnosis at early stages. The authors analyzed the datasets through ML algorithms and found that Random Forest Classifier is more effective than Support Vector Machine and k Nearest Neighbor for selected datasets. In this research, for every experiment, data were selected randomly 100 times to test the classification models. In this study, they found that the early identification of ASD is possible with a huge dataset; whereas, if the data sample is larger, the accuracy of diagnosis is also higher. Thus, the precision of Machine Learning based models would depend on the completeness of the data collected.

Thabtah et al. [35] suggested an application for screening autism spectrum disorder using mobile technology called ASDTests. This application contains tests in 11 different languages so that a large audience can participate. The application modules are designed to cater to distinct age groups including toddlers, children, teens and adults. Initially, this application served as a data collection tool and provided an ASD diagnosis. Professionals can use it to help people determine whether or not to pursue a formal clinical diagnosis. AQ-10 and Q-CHAT are used for screening in the application with visual aids.

Ruta et al. [36] employed a clinical sample collected in the Italian Healthcare system to authenticate the psychometric characteristics of the Q-CHAT questionnaire. Q-CHAT is a special test used to measure autism, not other brain conditions. In this study, 315 children participated. The authors compared autistic children (139) to those with developmental delay (50) and typically developing children (126). They also analyzed the statistical data related to these three groups. The scores on the Q-CHAT test were much higher in the autistic catagory compared to the groups with developmental delay and typically developing children.

Tartarisco et al. [37] employed ML algorithms to examine the precision of the Q-CHAT questionnaire in detecting autism in toddlers. In this study, they used a dataset of 265 children (*n* = 139 autistic children, *n* = 126 TD) collected by Ruta et al. [34] in three different Italian regions. The findings indicated that the Q-CHAT screening method has cross-cultural validity when used with an Italian sample. The authors concluded that Q-CHAT can be utilized in primary care settings, as it is a high-performance and easy-to-use tool. The study found that SVM is the best-performing ML model for their dataset.

Niedźwiecka et al. [38] used Q-CHAT to assess a sample of 1024 Polish children. This study aimed to identify ASD at an early age within a non-English speaking community and to investigate the association of symptoms with age, gender, or ASD family history. The research encompassed four distinct groups of participants: typically developing toddlers, toddlers whose parents reported concerns about ASD, toddlers experiencing delayed development, and elder siblings with ASD who are at risk for autism. The results showed that the number of boys with ASD is higher than that of girls, and age is not associated with Q-CHAT score.

Farooqi et al. [39] have discussed the challenges faced during the data collection process in a country such as Pakistan, where ASD cases are neither systematically tracked nor officially reported. The only government-run Child Psychiatry Department in Pakistan, Mayo Hospital, Lahore, provided a sample of 100 people with ASD. Data was gathered on the basis of a questionnaire with 21 questions. After applying an ML model to their dataset, the authors concluded that firstborns and males are more affected by ASD. There was no physical difference between autistic and non-autistic children. However, genes also play an important role, as 50% of children with ASD had some family members with a history of cognitive disability.

Jacob et al. [30] used the AUTOML-based tool Just Add Data Bio (JADBio) in their study on a dataset from the UCI repository. The data was initially collected by Thabtah [36] and is publicly available. In order to predict ASD, this study is the first to describe feature signatures and their importance in differentiating between classes.

AUTOML tools help make machine learning easier, but each works differently. autoSKLearn is good at tweaking settings for better results, but it might struggle with really big data. DAGAI uses special diagrams to build machine learning steps automatically, needing someone good at understanding these diagrams. Smart-ML is user-friendly with a nice visual interface, but it might not handle large amounts of data smoothly. H2O AUTOML covers many ways of performing machine learning but needs special tools. MLBox is simple but might need tweaks for specific needs. The TPOT is good for customizing but might take longer for complex tasks. Overall, these tools make machine learning easier, but each has its strengths and limitations, depending on the task [40].

The TPOT impulsively selects and enhance machine learning algorithms using genetic programming. Unlike AutoSKLearn, which employs Bayesian optimization for streamlining planning, the TPOT explores numerous approaches for optimizing machine learning algorithms. This versatility proves invaluable when dealing with intricate data relationships. The process is analogous to exploring various approaches to identify the most effective method for managing intricate datasets, and the TPOT demonstrates exceptional proficiency in this endeavor [41].

We have compiled the literature review by delineating the principal discoveries and constraints outlined in each paper, as shown in Table 1. Few researchers mentioned the limitations of their work, while some were identified by the authors by comparing the work with existing studies.

## 3. Proposed Framework

**Figure 1:**
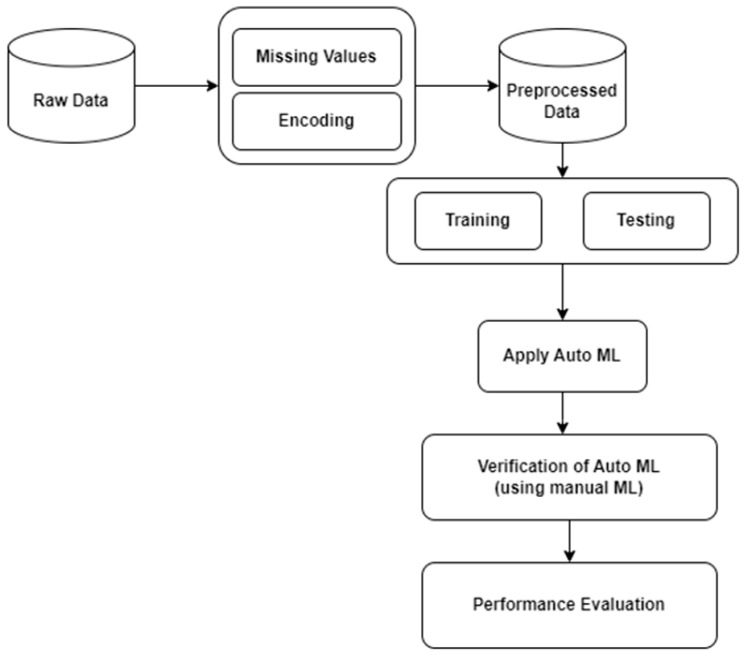
Workflow of the proposed system.

This section outlines a research framework that utilizes AUTOML techniques for the diagnosis of ASD in youngsters. There are various steps involved in our research process. Initially, data is collected and then preprocessed to refine it for the model. The data is then split into a training dataset and a testing dataset. Then, the model is trained using AUTOML, verification of the model is generated by AUTOML, and finally, a performance evaluation of the proposed AUTOML pipelines is conducted. Figure 1 shows the procedure of our system. A brief description of each step is discussed further.

### 3.1. Data Collection

We collected the data from several rehab centers in Pakistan. A questionnaire was circulated among the trainers and parents of children with autism. The responses received were stored in textual form in a Google sheet. It was a challenging process, as there is no proper reporting and tracking of ASD cases in the country. A questionnaire used to collect data was created based on the Q-CHAT screening method, because the data had to be recorded from scratch, in both hardware and software. Google Forms were used to collect data from online respondents. The responses received via Google Forms were downloaded in CSV format.

Our dataset originates from the Quantitative Checklist for Autism in Toddlers (Q-CHAT), which was developed by Allison et al. [47] for ASD detection. Later, Allison et al. proposed Q-CHAT-10 [48], which consisted of only 10 items. This shorter version of Q-CHAT showed the best results in previous studies, which is why we selected Q-CHAT-10 for our study, as shown in Table 2. Q-CHAT-10 also focuses on the most important questions for identifying ASD, as determined by statistical analysis of a larger set of questions. The illustrartion of the dataset is shown in Table 3. The data type of dataset variables a1–a10 is binary (0, 1).

The dataset consisted of 750 participants. The children were between 2 and 4 years old. Out of the total children, 434 were male and 308 were female. The questionnaire was filled in by either the parents, caregiver, medical staff, or clinician for each child.

To confirm the quality and relevance of the dataset, specific inclusion and exclusion criteria were applied. Children aged 2 to 4 years participated in the study. if their questionnaire responses were complete and accompanied by informed consent from a parent or legal caregiver. Participants were excluded if their responses were incomplete or if the child had a known comorbid neurological disorder in order to maintain focus on ASD-related traits without potential confounding conditions.

### 3.2. Data Preprocessing

Data preprocessing is the procedure of polishing raw or unorganized data to ensure its suitability for examination. This process entails purifying the data to remove any inconsistencies or errors. Firstly, we identified and removed missing/null values in our data. Since our dataset had categorical attributes, we encoded the data attributes such as sex, jaundice, and ASD class/traits into binary form 0 and 1. We eliminated the irrelevant attributes, such as “case_#” and “who completed the test”.

To evaluate the relationship between individual questionnaire responses and ASD diagnosis outcomes, we applied a Pearson correlation matrix. This statistical method measures the linear correlation between two variables—in this case, each of the ten Q-CHAT items (a1–a10) and the binary ASD class label (0 = non-ASD, 1 = ASD). The resulting correlation coefficients helped identify which features were most strongly associated with ASD traits. Features with low correlation were considered less informative for the predictive model, while highly correlated items were reviewed to avoid redundancy and multicollinearity. This process guided the selection and refinement of input features for model training.

**Figure 2:**
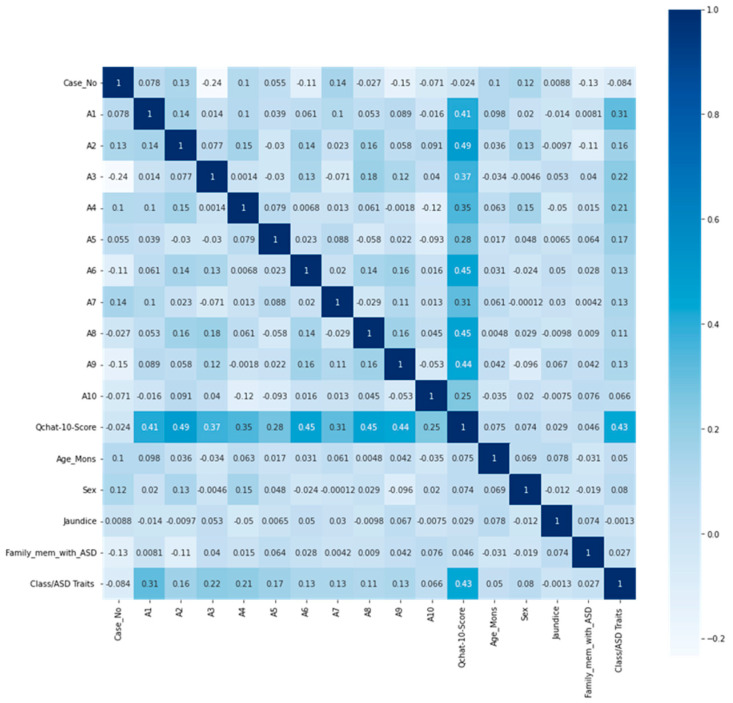
Correlation matrix of the dataset.

In Figure 2, A1, A2, A6, A8, and A9 were found to have a high correlation with Q-CHAT-10 score; thus, they were excluded from the dataset to improve the model’s capacity to generalize to novel data, simplifying it and minimizing repetition.

In this study, the primary statistical analysis conducted was a Pearson correlation investigation to evaluate the internal steadiness and trustworthiness of the Q-CHAT-10 questionnaire. This reliability assessment was essential to ensure the quality of input data used for model development. Since the core objective was to evolve a forecasting model for early ASD detection, the dataset was structured specifically for binary classification tasks. Each Q-CHAT-10 item was encoded as binary (yes = 1, no = 0), aligning with the modeling requirements of machine learning algorithms. Given this predictive focus, additional descriptive or inferential statistical analyses of the questionnaire responses were not performed, as they were beyond the intended scope of the study.

### 3.3. Data Partitioning 

The data partitioning method is a widely utilized technique to analyze the accomplishment of machine learning algorithms in prediction-oriented tasks. This method involves partitioning the dataset into two subsets: the training set and the test set. Typically, 80% of the data is used for the training pattern, with the outstanding 20% reserved for the test pattern. To train an automated machine learning (AUTOML) tool, we utilized 80% of the data (*n* = 593) as the training set, while the remaining 20% (*n* = 149) was set aside for testing the model’s accuracy and efficiency on unseen data. Through the random allocation of data into training and testing sequences, we conducted an assessment to determine whether our model exhibited overfitting or underfitting.

### 3.4. Model Development (AUTOML)

Uncovering the best ML algorithm and pipeline for a particular complication is not an easy task. It requires a lot of effort to collect and prepare the dataset. However, the result of the experiments is greatly influenced by the choice of machine learning technique and its associated pipeline.

**Figure 3:**
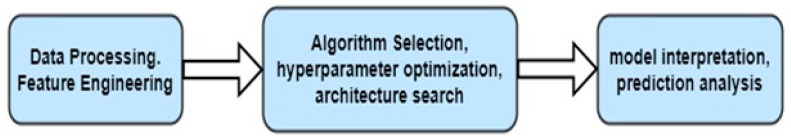
Workings of autoML.

The need for ML specialists is greater than the supply. To close this gap, progress has been made in creating user-friendly ML software that both novices and professionals can use. In this study, we used Python 3.9 on Google Colab with the TPOT AUTOML library (version 0.11.7) to develop and evaluate the predictive model. The ML workflow involves a substantial portion of automatically training and tuning multiple models within a user-defined time limit. The goal of AUTOML is to relieve data scientists from the burden of tedious and time-consuming operations (such as designing machine learning pipelines and optimizing hyperparameters) so they can focus more effectively on tasks that are considerably more challenging to automate. AUTOML solutions aim to automate some or all steps of the ML process, which includes the three common stages shown in Figure 3.

### 3.5. Tree-Based Pipeline Optimization Tool (TPOT)

**Figure 4:**
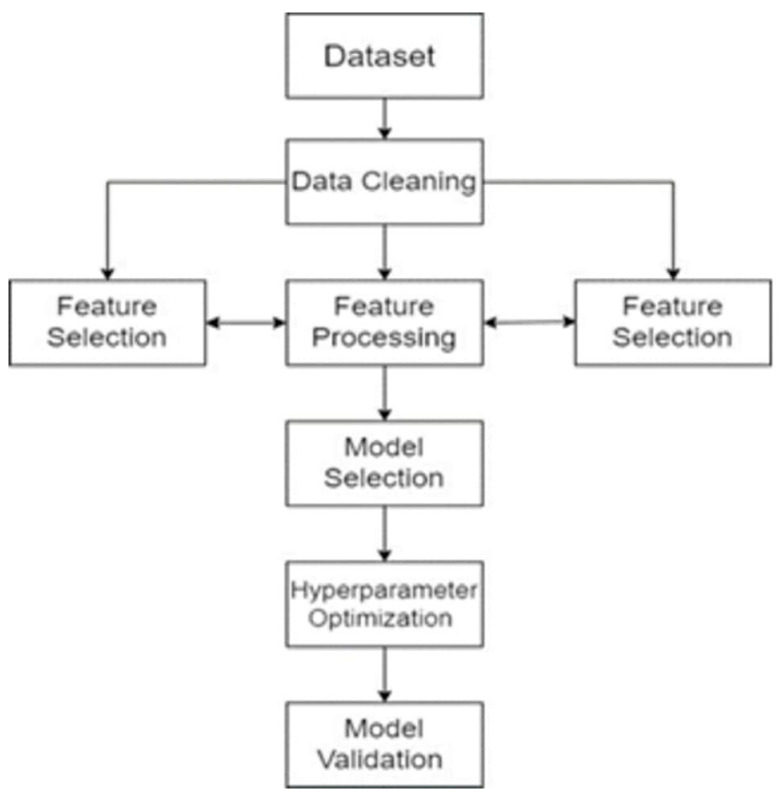
TPOT framework.

In analyzing our dataset, we employed the TPOT, which is a Python 3.9 library for AUTOML, available as open-source. The TPOT streamlines the tasks of model selection and tuning in machine learning. It employs a tree-based strategy to increase the performance and efficiency of pipelines. Figure 4 illustrates the TPOT framework, while the subsequent subsection provides additional insights into the workflow of the TPOT.

#### 3.5.1. Data Preparation

The TPOT initiates its process by preprocessing the input data. This encompasses various tasks, including handling missing values, coding categorical variables, and scaling quantitative characteristic and implementing any other essential data transformations required to ready the data for modeling.

#### 3.5.2. Feature Engineering

Following that, the TPOT might engage in feature engineering, which involves generating novel features or modifying current ones. These techniques encompass crafting interaction terms, incorporating polynomial features, or implementing dimensionality reduction approaches, like Principal Component Analysis (PCA).

#### 3.5.3. Pipeline Generation

The TPOT employs genetic programming to produce a wide array of machine learning pipelines. These pipelines encompass an assortment of preprocessing procedures, feature enhancement methods, and machine learning algorithms.

#### 3.5.4. Model Selection and Hyperparameter Tuning

The TPOT employs cross-validation on a training dataset to assess every generated pipeline. It evaluates the performance of each pipeline using a predetermined evaluation metric like accuracy, precision, or recall. The TPOT then identifies the top-performing pipelines and refines their hyperparameters to enhance their performance. This process entails exploring a predefined search space of hyperparameters for each machine learning algorithm incorporated in the pipeline.

#### 3.5.5. Model Evaluation

Following hyperparameter tuning, the TPOT assesses the chosen pipelines on a distinct validation dataset to gauge their generalization capabilities. This process ensures that the selected pipelines excel with unseen data and can adapt to novel samples effectively.

#### 3.5.6. Final Model Selection

In the end, the TPOT chooses the most effective pipeline by assessing its performance against the evaluation metric and its results on the validation dataset. This pipeline embodies the refined machine learning model crafted by the TPOT, customized to suit the unique dataset and problem in focus.

### 3.6. Performance Evaluation Metrics

In this work, we evaluated a diverse range of performance assessment measures to gauge the usefulness of the proposed framework. These metrics dispenses treasures insights into the precision and predictive abilities of the classification model. The following evaluation measures were used to observe the performance of our framework

- Confusion Matrix
- Precision
- Recall
- F1-Score
- AUC-ROC Analysis
- Precision–Recall Curve

## 4. Results and Evaluations

### 4.1. Data Analysis

**Figure 5:**
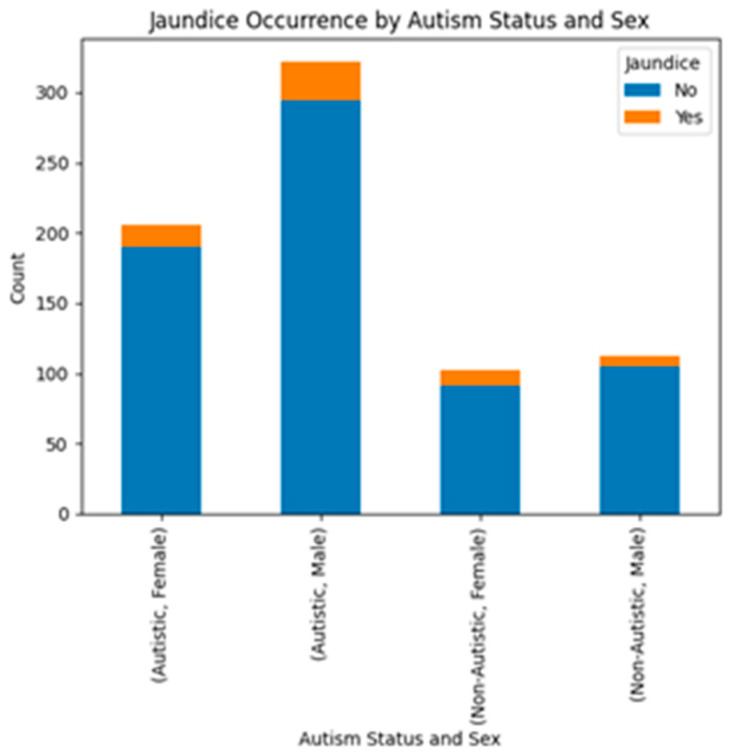
Visualization of jaundice occurrence by autism status and sex.

To visualize our dataset, we created several graphs. In Figure 5, we observed that the prevalence of jaundice was higher among male participants. However, statistical analysis using correlation methods showed no significant association between jaundice and ASD traits in our dataset. We also plotted the Q-CHAT-10 score by gender.

**Figure 6:**
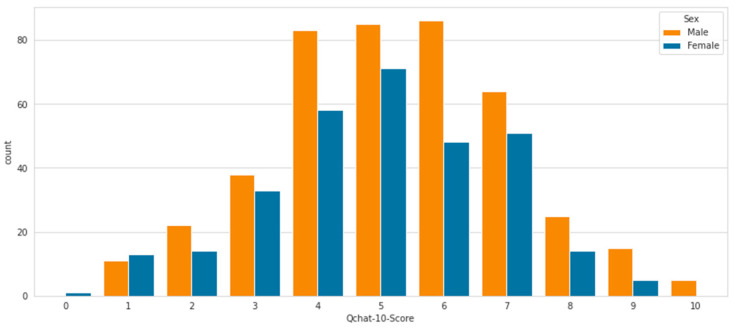
Visualization of the Q-CHAT-10 score according to gender.

We visualized the Q-CHAT-10 score according to gender. In Figure 6, we can see that male responses on the Q-CHAT have more positive answers than female responses. This indicates that males may be more prone to autism than females.

### 4.2. Experimental Results

We trained the AUTOML TPOT classifier by setting generations = 10 and population_size = 100, with random_state = 1 and k-fold cross-validation = 5, while keeping the other parameters as defaults. In the TPOT classifier, generations represent the number of repetitions performed by the genetic algorithm, and population size shows the number of individual pipelines generated and evaluated during the optimization process. Since the TPOT is a genetic programming-based optimizer that builds traditional machine learning pipelines, it does not involve neural network training, and thus parameters such as learning rate, epoch, and batch size are not applicable.

The best pipeline given by the TPOT was an ensemble of BernoulliNB and RandomForestClassifier, and the hyperparameters were optimized using TPOTClassifier. In this pipeline, the BernoulliNB classifier is used as the base model and the RandomForestClassifier is used as the meta model in the ensemble. The parameters for the best pipeline given by the TPOT are shown in Table 4.

In Support of non-autistic class, the precision was 0.68, indicating 68% of the instances forecasting as non-autistic were actually non-autistic. The recall was 0.53, meaning that 53% of the actual non-autistic instances were rightly forecasted as non-autistic. The F1-score for the non-autistic class was 0.60, which is the harmonic mean of the precision and recall for this class.

In case of autistic class, the precision was 0.83, indicating that 83% of the instances predicted as autistic were actually autistic. The recall was 0.90, meaning that 90% of the actual autistic instances were correctly predicted as autistic. The F1-score for the autistic class was 0.86, which is the harmonic mean of the precision and recall for this class.

The accuracy of the model was 0.78, which is the proportion of correctly classified instances out of all instances. The F1-score for each class was averaged to calculate the macro-average F1-score, resulting in a value of 0.73. On the other hand, the weighted average F1-score, which considers the number of instances in each class, was found to be 0.78.

Overall, the model has better performance on the autistic class than on the non-autistic class, as evidenced by the higher precision, recall, and F1-score for the autistic class. However, the performance of the model on the non-autistic class is still reasonable, with a precision of 0.68 and a recall of 0.53. The classification report for the AUTOML TPOT classifier is given in Table 5.

**Figure 7:**
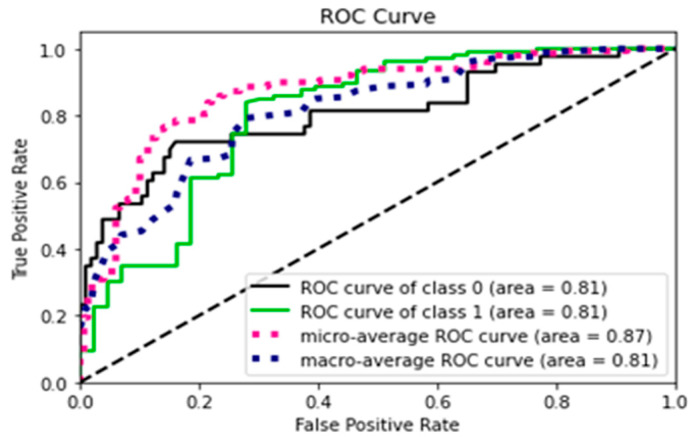
ROC curve for the experiment (AUTOML).

In Figure 7, the ROC curve analysis shows that the model has similar performance on both classes, as evidenced by the equal ROC scores for the autistic and non-autistic classes. The micro-average ROC score of 0.87 indicates that the model’s overall performance is good, with high true positive and low false positive rates. This value represents the overall performance of the model in terms of correctly identifying all instances regardless of the class label. The macro-average ROC score of 0.81 shows that the model’s performance is consistent across both classes.

**Figure 8:**
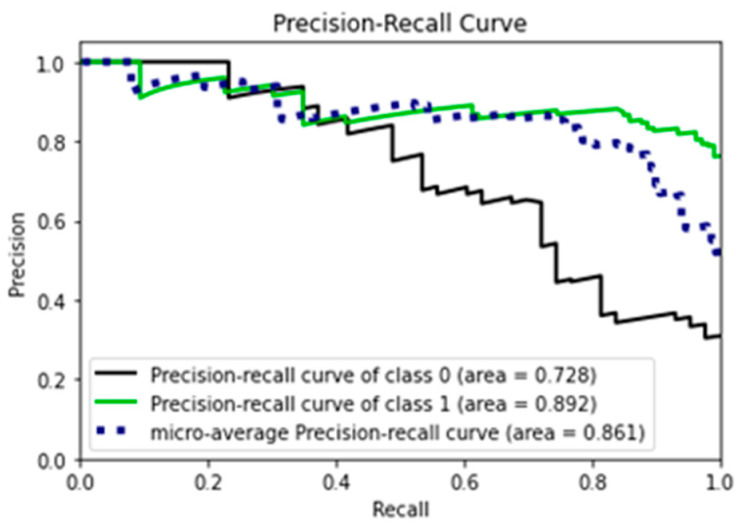
PR curve for the experiment (AUTOML).

In Figure 8, the precision–recall (PR) value for class 1, which is the autistic class, is 0.892, showing that the model correctly predicts instances as autistic 89.2% of the time. The PR value for the non-autistic class is 0.728, meaning the model correctly predicts instances as non-autistic 72.8% of the time.

The PR curve analysis shows that the model has better performance on the autistic class, as evidenced by the higher PR score. However, the model’s performance on the non-autistic class is still reasonable, with a PR score of 0.728. The micro-average PR score of 0.861 indicates that the model’s overall performance is good.

### 4.3. Verification of the Experiment

To verify the models generated by AUTOML we manually implemented the same model generated by AUTOML’s TPOT framework. For this purpose, the parameters given by the TPOT pipeline were used for the model’s implementation along with the TPOT classifier. The results from the verification of the experiment are shown in Table 6. Similarly, we obtained the same visualizations for the verification model as in the experiment.

## 5. Comparative Analysis

A comparison was conveyed out to analyze the performance of the AUTOML driven model relative to the traditional machine learning model. We compared it with support vector machine (SVM), k-nearest neighbor (KNN), naïve Bayes (NB), and logistic regression (LR). Table 7 shows the comparison of these models. For the performance evaluation of the models, we computed accuracy and a confusion matrix. Table 7 shows that our proposed AUTOML generated model performed superior than other classical Machine Learning models.

The proposed AUTOML model trains on the given dataset within a few minutes and predicts ASD, which is a much faster diagnosis process compared to manual diagnosis. So, our Artificial Intelligence (AI)-driven ASD detection framework will supplement manual diagnosis detection for identifying severity with clinical psychologists. AI detection and manual detection are interconnected in that AI utilizes the authentic diagnostic criteria of DSM-5-TR through ML, and it is more efficient regarding time, cost of treatment, and raising parental awareness through early detection. AI detection is a fast and accurate method that can detect the disorder within a few minutes, whereas manual diagnosis requires plenty of time and multiple visits to clinics for child observation and clinical interviews from parents or caregivers. AI detection reduces the expense of multiple pre-diagnosis visits to clinical psychologists and differentiates between intellectual disability disorder based on formed questions derived from the diagnostic criteria of DSM-5 TR.

## 6. Discussion

In this work, we investigated the performance of automated machine learning (au-toML) framework for detecting autism spectrum disorder (ASD) at early ages. The classifier was trained using the TPOT with 10 generations and a population size of 100 while keeping other parameters as default. The best pipeline generated by the TPOT consisted of an ensemble of BernoulliNB and RandomForestClassifier, optimized using TPOTClassifier.

Our findings suggest that the classifier demonstrated superior performance evaluation metrics for the autistic class compared to the non-autistic class. Specifically, precision, recall, and F1-score for the autistic class were 0.83, 0.90, and 0.86, respectively, whereas for the non-autistic class, they were 0.68, 0.53, and 0.60, respectively.

Furthermore, we noted a significant impact on the output of the TPOT library when increasing the generation and population size. Larger sizes enabled the TPOT to explore and execute more pipelines simultaneously, resulting in improved outcomes. However, it is essential to consider the longer processing times and increased computational demands associated with larger sizes.

In summary, utilizing AUTOML libraries, like the TPOT, necessitates an understanding of their computational needs and considerations regarding pipeline interpretability. The interpretability and reproducibility of TPOT’s results, coupled with the ability to customize pipelines and restrict ML algorithms, offer valuable tools for minimizing computational expenses while upholding performance standards.

A restriction of this study is the shortage of an evaluation on publicly available datasets. Since the dataset used in our study was locally collected from Pakistani rehabilitation centers, its demographic and cultural specificity may limit generalizability across international populations. Additionally, public datasets, such as the UCI ASD Screening Data, involve different socio-clinical contexts, which could introduce bias if compared directly.

## 7. Conclusions and Future Detection

In brief, the primary motive of this study was to develop a framework for diagnosing ASD utilizing AUTOML with our dataset. The objective is to enhance the precision of ASD diagnosis in children and expedite the process compared to conventional methods. For this purpose, data of children was collected using a survey based on Q-CHAT-10. We applied AUTOML TPOT to our dataset for ASD prediction and evaluated the outcomes of this technique. We used different evaluation metrics, including precision, recall, F1-score, and AUC-ROC curves, to evaluate the performance of our model, AUTOML. The results showed that the model performs better on the autistic class than the non-autistic class.

For future work, we see opportunities to expand and refine this study. We plan to explore the use of other AUTOML libraries to develop additional models and compare their performance. This comparative approach will help identify the most effective tools and techniques for early ASD diagnosis, potentially leading to more accurate and efficient screening methods. In future work, we aim to evaluate our model on publicly available ASD datasets, such as those from the UCI repository, to benchmark performance across diverse populations and validate its robustness in broader diagnostic contexts.

## Figures and Tables

**Table 1:** Summary of the existing studies.

Ref. Year	Key Findings	Limitations
[1] 2021	An automated model that supports medical professionals identify ASD was presented in this study.	The limitations are the unavailability of open-source datasets coupled to ASD.
[7] 2019	Three datasets for youngsters, teenagers, and grown-ups with ASD were employed to classify ASD using the SVM, KNN, and RF algorithms.	Unavailability of a complete dataset related to ASD.
[35] 2019	In this research, the ASDTests app was proposed for data collection and assisting health professionals in ASD detection.	It was not possible to conduct feature analysis using the app.
[36] 2019	An ML approach was used on a dataset collected by authors Q-CHAT with 25 items was evaluated.	The sample size was relatively small and not open-source.
[37] 2021	The study assessed multiple machine learning methods on four datasets to classify autism spectrum disorder. SVM and KNN emerged as the most effective techniques for accurate classification	The study did not investigate the influence of demographic factors (age, gender, race/ethnicity) on machine learning accuracy
[42] 2020	In this paper, the automated detection system using a convolutional neural network (CNN) achieved high accuracy in detecting ASD in children based on their functional magnetic resonance imaging (fMRI) data.	This study was conducted on a relatively small sample size of 40 participants (20 with ASD and 20 typically developing controls). Further, the study only used dormant-state fMRI data.
[43] 2022	In this study, various works on algorithmic approaches to classify ASD were presented, e.g., one study showed that the multichannel deep attention neural network (DANN) performed better than support vector machines (SVMs).	This study did not explore the detailed ethical implications of using algorithmic approaches in the diagnosis and treatment of ASD.
[44] 2022	This study showed automated methods. This study outperformed non-automated ones in accuracy, sensitivity, and specificity. Combining machine learning techniques enhanced ASD diagnosis accuracy.	This study only focused on facial images as a diagnostic tool and did not consider other potential sources of information
[45] 2020	The research revealed that employing automated machine learning techniques enables precise forecasting of brain age based on cortical anatomical measurements	The study was conducted on a proportionate small sample size
[41] 2016	The study found that the Tree-based Pipeline Optimization Tool (TPOT) autonomously outperforms basic machine learning without human input on benchmarks.	The study used supervised classification benchmarks; further research is needed to assess TPOT’s performance on different ML problems
[46] 2024	The study compares the AUTOML tools TPOT and KNIME for ASD detection.	The study did not compare other available AUTOML tools.

**Table 2:** Q-CHAT-10 description.

Dataset Variables	Description of Q-CHAT-10 DATASET FEATURES
α1	Does your child show attention by turning toward you when you call their name?
α2	How easily can you make direct eye contact with your child?
α3	Does your child use gestures to ask for things he wants, such as a toy that is out of his reach?
α4	Does your child point to objects or events to draw others’ attention or express excitement?
α5	Does your child engage in imaginative activities, such as pretending to feed a doll or talk to a toy telephone?
α6	Does your child follow another person’s gaze or look in the same direction when someone else is looking at something?
α7	When a family member seems upset or distressed, does your child attempt to comfort them, for example by offering physical contact or affection?
α8	How would you characterize your child’s early verbal communication?
α9	Does your child use simple nonverbal gestures, such as waving to say goodbye?
α10	Does your child often stare at objects or into space for extended periods without a clear purpose?

**Table 3:** Dataset description.

Dataset Variables	Data Type	Attribute Description
Age_Months	Number	Child’s age in months
Sex	String	Male/female
Jaundice	Boolean (Yes/No)	Whether the child was born with jaundice
Family_mem_with_ASD	Boolean (Yes/No)	Any family member diagnosed with ASD
Who completed the test	String	Parent, caregiver, medical staff, clinician
Qchat-10-Score	Integer	Final results based on the scoring function
Class/ASD Traits	Boolean	The class label represents whether ASD-related traits are observed, with a value of ‘0’ indicating no traits present and ‘1’ indicating that such traits are present

**Table 4:** Parameters for the best pipeline.

TPOT Best Pipeline Parameters	Values
Alpha	1.0
fit_prior	False
Bootstrap	True
Criterion	gini
max_features	0.05
min_samples_leaf	1
min_samples_split	4
n_estimators	100

**Table 5:** Classification report of the experiment (AUTOML).

Experiment	Precision	Recall	F1-Score	Support
Non-autistic	0.68	0.53	0.60	43
Autistic	0.83	0.90	0.86	106
Macro avg	0.75	0.72	0.73	149
Weighted avg	0.78	0.79	0.78	149
Accuracy	-	-	-	0.83

**Table 6:** Classification report—verification of the experiment (manualML).

Verification	Precision	Recall	F1-Score	Support
Non-autistic	0.68	0.53	0.60	43
Autistic	0.83	0.90	0.86	106
Macro avg	0.75	0.72	0.73	149
Weighted avg	0.78	0.79	0.78	149
Accuracy	-	-	-	0.83

**Table 7:** Comparison with ML models.

Model	Accuracy	Precision	Recall	F1-Score
Support Vector Machine	0.71	0.718	1.000	0.836
K-Nearest Neighbour	0.74	0.785	0.888	0.833
Naive Bayes	0.80	0.847	0.887	0.866
Logistic Regression	0.757	0.805	0.875	0.838
Decision Tree	0.70	0.804	0.769	0.786
Random Forest	0.780	0.821	0.888	0.854
AUTOML	0.83	0.868	0.868	0.868

## Data Availability

The original contributions presented in the study are included in the article, further inquiries can be directed to the corresponding author.
